# Proliferative effect and osteoinductive potential of extracellular matrix coated on cell culture plates

**DOI:** 10.1186/2193-1801-2-303

**Published:** 2013-07-05

**Authors:** Yong Guo, Qiangchen Zeng, Yuxian Yan, Liang Shen, Lu Liu, Ruixin Li, Xizheng Zhang, Jimin Wu, Jing Guan, Shujie Huang

**Affiliations:** Institute of Medical Equipment, Academy of Military Medical Sciences, Tianjin, 300161 China; Biology department, Shandong provincial Key laboratory functional macromolecular biophysics, Dezhou University, Dezhou, 253021 China; Center Lab, Logistics University of Chinese Peoples Armed Police Forces, Tianjin, 300162 China; Tianjin Institute of Medical Equipment, No.106 Wangdong Road, Hedong District, Tianjin, 300161 China

**Keywords:** Extracellular matrix, Bone marrow-derived mesenchymal stem cells, Osteoblasts, siRNA, Proliferation, Differentiation

## Abstract

Different cell/tissue derived extracellular matrix (ECM) display subtle differences that might provide important cues for proliferation and differentiation of cells in vitro or in vivo. However, the bioactivities of different ECMs in vitro were not fully understood. In this study, osteoblasts-derived and fibroblast-derived ECM-coated cell culture dishes were prepared respectively by culturing osteoblastic MC3T3-E1 cells and rat fibroblast then decellularizing the cultures. We investigated the bioactivities of the two different ECMs coated on cell culture plates using cellular, biochemical and molecular method. The proliferative activity of the bone marrow-derived mesenchymal stem cells (BMSCs) cultured on osteoblast-ECM was lower than for BMSCs grown on fibroblast-ECM. Compared with the BMSCs cultured on fibroblast-derived ECM, the cells grown on osteoblastic ECM showed enhanced alkaline phosphatase (ALP) activity, higher BMP-2 and osteopontin protein levels, increased secreted calcium content, and higher levels of runt-related transcriptional factor 2 (Runx 2) and osteocalcin (OCN) mRNA. Knockdown of BMP-2 or FGF-2 with shRNA transfection hardly effected osteoblastic differentiation or proliferation of MC3T3-E1 seeded on osteoblast-ECM or fibroblast-ECM. Therefore, the osteoblastic ECM had better osteoinductive potential and lower proliferative effect than fibroblastic ECM, and the two ECM presented enough bioactivity, knockdown of growth factors had no significant effect on differentiation and proliferation of re-seeded cells.

## Background

Extracellular matrix (ECM) secreted by the resident tissue cells contains various protein fibers interwoven into a hydrated gel composed of a network of glycosaminoglycan chains (Nelson and Tien 2006; Badylak 2007, Badylak et al. 2009). ECMs have been established as potent regulators of cell function and differentiation (Adams and Watt 1993; Badylak 2005). It provides mechanical support to the growing cells and affects cell adhesion, proliferation, differentiation, morphology, and gene expression (Kleinman et al. 2003). In principle, the ECM best represents the native cellular and tissue microenvironment (Discher et al. 2009).

In recent years, a considerable effort has been put into the research on *in vitro* bioactivity of ECM coated on culture plates. After the cells grown in culture plates are removed using chemical or physical methods, the bioactivity of the resultant ECM coated on the dishes can be easily investigated. The osteogenic cell (MC3T3-E1)-derived ECM, attached to the plates, promotes the osteogenic differentiation of embryonic stem cells seeded on the ECM (Evans et al. 2010). It has been demonstrated that cardiac fibroblast-ECM supports early maturation of ES-derived cardiomyocytes *in vitro* (Baharvand et al. 2005), and improves proliferate and cellular adhesion of BMSCs (Sreejit and Verma 2011).

Cell-derived ECM has been fabricated into three-dimensional scaffolds or reconstituted with scaffolds for tissue engineering applications (Liao et al. 2010; Wolchok and Tresco 2010; Lu et al. 2011). It has been shown that osteoblastic ECM deposited on titanium fiber mesh scaffolds induces osteoblastic differentiation of mesenchymal stem cells (MSCs) in static culture (Datta et al. 2005). In three-dimensional scaffold, osteoblast-ECM promotes osteogenic differentiation of embryonic stem cells and improves osteoblastic differentiation of marrow stromal cells (Datta et al. 2005; Pham et al. 2008), and supports adhesion, growth, and ECM production of osteoblasts *in vitro* (Tour et al. 2011).

ECM provides supportive microenvironment for mammalian cells *in vitro*. The ECMs of different tissue types display subtle differences that might provide important cues for various resident cells; it has been shown that tissue-specific extracellular matrices promote tissue-matched cell proliferation and maintenance of cell phenotype (Zhang et al. 2009). ECM serves as a reservoir of growth factors and cytokines, such as BMP, fibroblast growth factor, and vascular endothelial growth factor; these key molecules bind to either polysaccharide or protein constituents of ECM (Suzawa et al. 1999; Pike et al. 2006; Faure et al. 2008). These bioactive proteins/peptides binding to ECM regulate cell proliferation and differentiation, which suggests that ECMs produced in vitro display bioactivities, such as proliferative effect and inductive potential. However, the in vitro bioactivities of various ECMs were not fully understood.

This study was carried out to compare the bioactivities of two representatively different ECMs: osteoblast-derived ECM and cardiac fibroblast-derived ECM. The two ECMs which were coated on cell culture plates, were prepared respectively. Then, BMSCs or MC3T3-E1 pre-osteoblastic cells were cultured on the plates covered with the different ECMs, and these cells were examined for proliferation and osteogenic differentiation respectively.

## Material and methods

This study was approved by The Institutional Animal Care and Use Committee of Tianjin Institute of Medical Equipment. All experimental animals were cared for according to the Guide for the Care and Use of Laboratory Animals published by National Institute of Health of USA.

### Isolation and culture of cardiac fibroblasts

Cardiac fibroblasts from ventricles of neonatal C57BL/6 mouse hearts were isolated and cultured according to a well-established method (Pan et al. 1999). Briefly, hearts from neonatal mice were removed, minced and trypsinized at 37°C with gentle stirring in PBS containing 0.25% trypsin (Invitrogen). Then, the cells were centrifuged and resuspended in complete α-MEM medium supplemented with 10% fetal calf serum and 1% penicillin-streptomycin (Invitrogen). After incubation at 37°C for 45 min, the attached cells (most of them were cardiac fibroblast) were prepared. After 3 passages of subculture, over 95% of cells were identified as cardiac fibroblasts by anti-α-sarcomeric actin and anti-vimentin antibody immunostaining.

### Preparation of osteoblast-ECM and cardiac fibroblast-ECM

Osteoblasts were derived from MC3T3-E1 cells (clone 4, ATCC catalogue number CRL-2593; ATCC Teddington, UK), a mouse monoclonal pre-osteoblastic cell line that has been shown to differentiate into osteoblasts and osteocytes (Sudo et al. 1983; Franceschi and Iyer 1992). For differentiation, the cells grown to confluence were maintained for further 8 days in complete a-MEM medium supplemented with 10% fetal calf serum and 1% penicillin-streptomycin and in the presence of 280 μmol/L ascorbic acid, 10 mmol/L β-glycerophosphate and 0.1 μmol/L dexamethasone (Sigma). Cardiac fibroblasts grown to confluence were maintained for further 8 days in complete a-MEM medium supplied with 280 μmol/L ascorbic acid.

The cells were removed according to an established method (Shirasuna et al. 1993), with some modifications. Briefly, the cultures of cardiac fibroblasts and osteoblasts were washed with PBS respectively, the cells were removed by incubation for 3 min with PBS supplemented with 0.5% Triton X-100 and 10 mmol/L NH_4_OH, and then washed three times with PBS. The ECMs attached to the dishes were treated with 100 units/ml DNase (Sigma-Aldrich, St. Louis, MO, USA) for 1 h, and the resulting ECMs were rinsed with PBS, observed by inverted microscopy. Then the plates coated with ECM were stored at 4°C. Some of the ECMs coating the wells were fixed with 3% paraformaldehyde, and stained with Van Gieson (VG) Histochemical Staining Kit (Nanjing Jiancheng Biotechnology Co. Ltd, China, containing hematoxylin solution) to dye the collagen fibers red, and nuclei blue. The plates or wells without ECM were the control groups in the experiment illustrated below.

### Cellular adhesion assays

BMSCs were seeded into ECM-coated 24-well cell culture plates coated with different ECMs, allowed to adhere for 2 h at 37°C, then washed gently with PBS to remove non-adherent cells; the adherent cells were not confluent. After incubation with 0.5mg/ml MTT (3-(4, 5-dimethylthiazol-2-yl)-2,5-diphenyltetrazolium bromide, Promega, USA) solution provided (0.30 mL/well)) for two hours at 37°C, the cells were washed with PBS, and 0.30 mL acidic isopropanol (0.04 mol/L M HCl in absolute isopropanol) was added to each well; the plates were shaken to dissolve the converted dye completely. Then the absorbance was measured at 570 nm on an enzyme-linked immunosorbent assay reader. The relative adhesion potential of BMSCs on ECM was expressed as absorbance at 570 nm (Abs570nm).

### Proliferation assays

The BMSCs (Cyagen Biosciences, USA) of passage four, 1.0 × 10^4^ per well, were plated into 24-well cell culture plates coated with different ECMs. The MTS CellTiter 96^®^ AQueous One Solution cell proliferation assay kit (Promega, Madison, USA) was used to assay for living cells, according to the instructions of the manufacturer. After incubation for 1.5 h at 37°C, the plates were read on an enzyme-linked immunosorbent assay reader at 490 nm. The absorbance at 490 nm (Abs490nm or OD490nm) was used as a measure of living cell population size; the quantity of MTS (3-(4,5-dimethylthiazol-2-yl)-5-(3-carboxymethoxyphenyl)-2- (4-sulfophenyl)-2H-tetrazolium, inner salt) formazan product expressed as OD490nm is directly proportional to the number of live cells.

### ALP activity assay

After trypsinization and centrifugation, the cells were lysed by brief sonication on ice in a lysis buffer (10 mmol/L HEPES, 250 mmol/L sucrose, 5 mmol/L Tris–HCl, 0.1%TritonX-100, pH 7.5). The ALP activity of the lysates was assayed with ALP Activity Assay Kit (Nanjing Jiancheng Biotechnology Co. Ltd, China) at 25°C, according to the manufacturer’s protocol. ALP activity of each sample was normalized to protein concentration.

### Measurement of extracellular deposition of calcium

Calcium deposition in the 24-well plates was measured using a calcium assay kit (Nanjing Jiancheng Biotechnology Co. Ltd, China). Briefly, after removal of the BMSCs seeded in the plates coated with different ECMs, the wells of the plate were washed with deionized distilled water, and then incubated overnight in the presence of 0.5 M acetic acid. A standard curve was generated using serial dilutions of CaCl_2_, and the calcium deposited was quantified as μg Ca^2+^ equivalent per well. Before re-seeding the cells, the calcium of the coated ECM in each well of cell culture plate was assayed. The calcium deposition content of the ECM without re-seeded cells was subtracted, then the remnant calcium content was secreted by the BMSCs which seeded on the ECMs.

### Western blot analysis of BMSCs

After centrifugalization, cell lysates were harvested using RIPA lysis buffer containing protease inhibitors (pH 7.4, 50 mM Tris, 150 mM NaCl, 1% NP-40, 0.1% sodium dodecyl sulfate, 0.5% sodium deoxycholate, and protease inhibitors in the buffer: 0.5 μg/ml leupeptin, 1 mM ethylenediaminetetraacetic acid, 1 mM Na3VO4, 1 mM phenylmethylsulfonyl fluoride, 2 mM sodium pyrophosphate and 25 mM β-glycerophosphate) (Beijing Biomed Co. Ltd, China). The protein concentration of the lysates was measured according to bicinchoninic acid assay (BCA) method, and equal amounts of proteins were separated by sodium dodecyl sulfate polyacrylamide gel electrophoresis and electro-transferred onto polyvinylidene difluoride membranes (Millipore, Bedford, MA, USA). After blocking with 5% skim milk, the membranes were incubated overnight respectively with the mouse anti-osteopontin (SC21742, 1:500; Santa Cruz, Santa Cruz, USA) and rabbit anti-BMP-2 (BA0585, 1:500; Boster, Wuhan, China), then washed with PBS and incubated with horseradish peroxidase-conjugated secondary antibody. The immunoreactive bands were visualized using an enhanced chemiluminescence detection kit (Santa Cruz Biotechnology, Santa Cruz, CA, USA). Optical density of the protein bands was determined with Gel Doc 2000 (Bio-Rad, CA, USA). The expression of glyceraldehyde3-phosphatedehydrogenase (GAPDH) of the cells was used as a loading control, data were normalized against those of corresponding GAPDH.

### Knockdown of BMP-2 or FGF-2 with small hairpin RNA (shRNA) interfering

Small interfering RNA targeting mouse BMP-2 (shBMP2) and FGF-2 (shFGF2) was purchased from Santa Cruz Biotechnology (Santa Cruz, CA, USA). MC3T3-E1 pre-osteoblastic cells (60%-70% confluence) were transfected with shBMP2 (shFGF2) or control shRNA (shCtl) using High-Fect Transfection Reagent (Beijing Cowin Bioscience Co., Ltd., Beijing, China), according to the manufacturer’s recommendations. Two days after transfection, following experiments (ALP activity assay, Western blot, real-time PCR and proliferation assays) were performed at indicated time.

### Real-time polymerase chain reaction (PCR)

Total RNA was extracted with Trizol (Invitrogen, USA), then used for the first strand cDNA synthesis using Rever TraPlus Kit (TOYOBO, Japan). Real time PCR was performed to determine the mRNA levels of runt-related transcriptional factor 2 (Runx 2), osteocalcin (OCN) and glyceraldehyde3-phosphatedehydrogenase (GAPDH, a housekeeping gene) was used as an internal control. The reaction was conducted using SYBR Green I PCR Mix (Invitrogen) on an Applied Biosystems 7900 Real Time PCR System, according to the manufacturer’s instructions. Primer sequences are listed in Table 1. The amplification reaction included three steps, (1) 94°C for 3 min; (2) 94°C for 15 s; and (3) annealing and extension at each annealing temperature for 30 s. The steps 2 and 3 were repeated for 40 cycles. Using the relative quantitative method (2^-ΔΔCt^), the expression levels of the PCR products of interest relative to those in the control group were calculated.

**Table 1:** **Primers used for Real-Time PCR analysis**

Gene	Primer sequence (5′-3′)	length(bps)
Runx2	F:AGTAGCCAGGTTCAACGAT	90
	R:GGAGGATTTGTGAAGACTGTT	
OCN	F:AGTCTGACAAAGCCTTCA	134
	R:AAGCAGGGTTAAGCTCACA	
GAPDH	F: ACCCATCACCATCTTCCAGGAG	159
	R: GAAGGGGCGGAGATGATGAC	

### Statistical analysis

The data were presented as mean ± standard deviation, and analyzed by SPSS10.0 program (Chicago, IL, USA). One-way analysis of variance (ANOVA) with a post-hoc test was performed, and the statistical differences between the two groups were determined by the Student’s t test. A value of P < 0.05 was considered statistically significant. All experiments were repeated at least 3 times, and representative experiments are shown.

## Results

### ECM preparation

**Figure 1:**
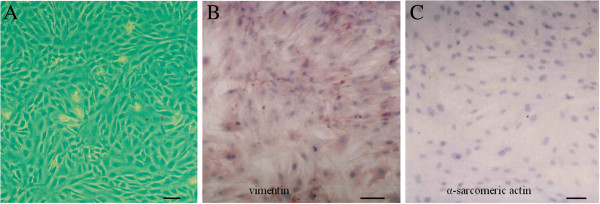
**Cardiac fibroblasts were identified.** Cardiac fibroblasts in vitro were observed **(A)** and identified using anti-vimentin **(B)** and anti-α-sarcomeric actin **(C)** antibody immunostaining. (bar: 100 μm).

The results of immunostaining showed that the isolated cells were positively stained with anti-vimentin antibody and negatively stained with anti-α-sarcomeric actin antibody (Figure 1). The result indicated that the cells we cultured were cardiac fibroblasts.

**Figure 2:**
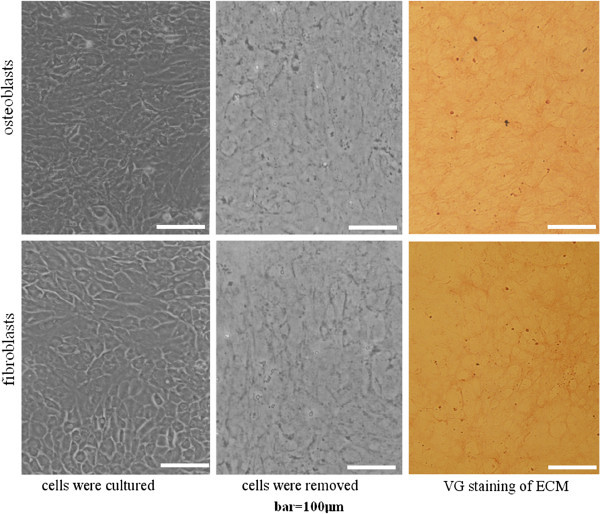
**Preparation and VG histochemical staining of osteoblast-ECM and fibroblast-ECM.** Osteoblasts and fibroblasts were removed after treatment with PBS containing 0.5% Triton X-100 and 0.10 M NH_4_OH, and the ECMs formed on the surfaces were revealed. VG staining with VG kit containing hematoxylin solution showed collagen fibers which were stained red and no nuclei. (bar: 100 μm).

After treatment with PBS containing 0.5% Triton X-100 and 0.10 M NH_4_OH, all the cells were removed, leaving behind the ECM. Under inverted microscope, the osteoblast- and fibroblast-ECM looked the same (Figure 2). VanGieson staining results (with VanGieson Kit containing hematoxylin solution) showed no visible nuclei but large numbers of collagen fibers could be seen in both types of ECM (Figure 2). No difference was found between the staining results.

### BMSCs proliferation and adhesion assays

**Figure 3:**
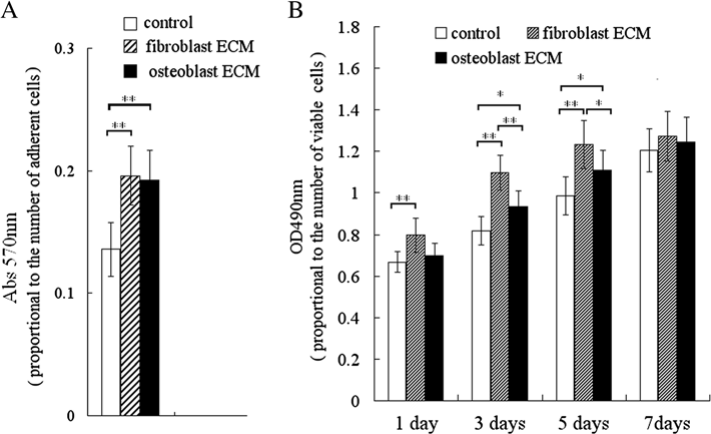
**Assay of relative adhesion potential and proliferation of BMSCs grown on osteoblast-ECM and fibroblast-ECM. A**: the relative adhesion potential was assayed with MTT reagent, the results indicated that the adhesion potential of BMSCs grown on cardiac fibroblasts-ECM was nearly the same as on osteoblast-ECM. **B**: The proliferative acticity was assayed with MTS reagent, the results indicated the proliferation of BMSCs was the highest on the fibroblast-ECM, and the proliferation on osteoblast-ECM was higher than control group. * P <0.05, **P <0.01, between the indicated groups, n = 7 per group, control group, no ECM coated.

The results of cellular adhesion assays showed that the adhesion potential of BMSCs in osteoblast-ECM was nearly the same as in cardiac fibroblast-ECM (Figure 3A).

The proliferation activity was examined using the MTS assay and expressed as OD490nm. The results of the MTS assay indicated that the proliferative effect of cardiac fibroblast-ECM was stronger than that of osteoblast-ECM and control group (without ECM coating), and the proliferative effect of osteoblast-ECM was stronger than control group (Figure 3B).

### Osteoblastic differentiation of BMSCs seeded on ECMs

**Figure 4:**
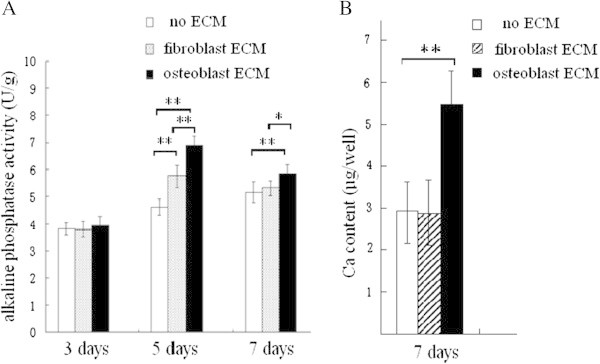
**Alkaline phosphatase (ALP) activity and extracellular calcium (Ca) deposition of BMSCs seeded on the two different ECMs.** ALP activity was detected using the p-nitrophenyl phosphate method. After 5, 7 days of culture, the result showed that the ALP activity of BMSCs grown on osteoblast-ECM is higher than for other groups **(A)**. After 7 days of culture, the Ca content was assayed using the methyl thymol blue complexon method, the result indicated that BMSCs grown on osteoblast-ECM secrete more Ca than the cells in other groups **(B)**. **P <0.01, * P <0.05, between the indicated groups, n = 7 per group.

To investigate whether the ECMs could stimulate osteoblastic differentiation of BMSCs, the cells were seeded on ECM-coated plates. ALP activity assay was performed after 3, 5 and 7 days of incubation, and extracellular calcium content assay was performed after 7 days. The results showed that the ALP activity in the cells grown in osteoblast-ECM coated plates was higher than in the cells cultured with cardiac fibroblast-ECM and in the control group (Figure 4A).

After 7 days of growth, the calcium deposition of the cells cultured on osteoblast-ECM coated plate was more substantial than in other two groups (Figure 4B).

**Figure 5:**
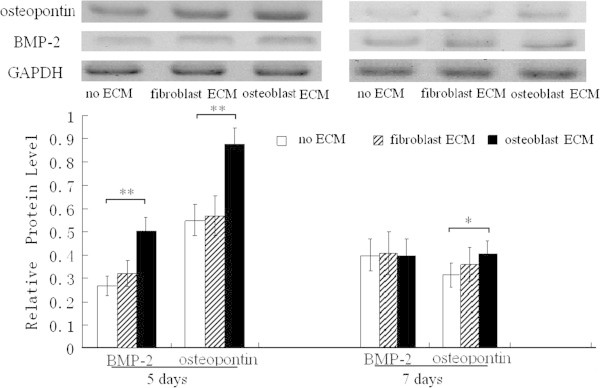
**Western blot analysis of BMP-2 and osteopontin in BMSCs seeded on the ECMs.** After 5, 7 days of culture, the relative protein expression levels of osteopontin (normalized to GAPDH) are higher in BMSCs seeded on osteoblast-ECM than in other groups. After 5 days of culture, the protein level of BMP-2 is higher than other group. * P <0.05, **P <0.01, between the indicated groups, n = 5 per group.

The immunoblotting data demonstrated that osteopontin protein expression levels of BMSCs seeded on osteoblast-ECM were the highest in the three groups (after 5 days of culture, Figure 5). The BMP-2 level was the highest 5 days later (Figure 5).

**Figure 6:**
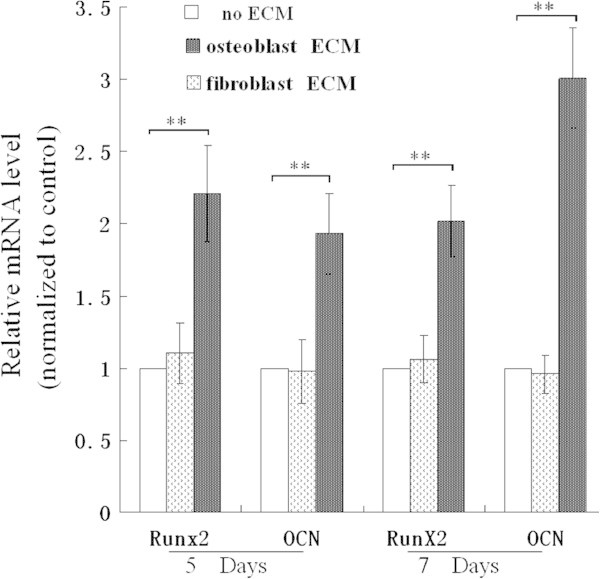
**mRNA expression levels of Runx2 and OCN in BMSCs seeded on the ECMs.** After 5 and 7 days of culture, the relative mRNA level was assayed using real time polymerase chain reaction, Runx2 mRNA and OCN mRNA are the highest levels in BMSCs seeded on osteoblast-ECM, * P <0.05, **P <0.01, between the indicated groups, n = 6 per group.

The levels of Runx2 and OCN mRNA in BMSCs seeded on osteoblast-ECM were both higher than in the cells grown on cardiac fibroblast-ECM and in the control group (after five and seven days of growth, Figure 6).

In addition, the osteoinductive potential of cardiac fibroblast-ECM was very limited, it is nearly as low as control group without ECM coating (Figures 4, 5, 6).

### Proliferative activity or osteoblastic differentiation of MC3T3-E1 cells transfected with shBMP2 or shFGF2 and re-seeded on ECMs

**Figure 7:**
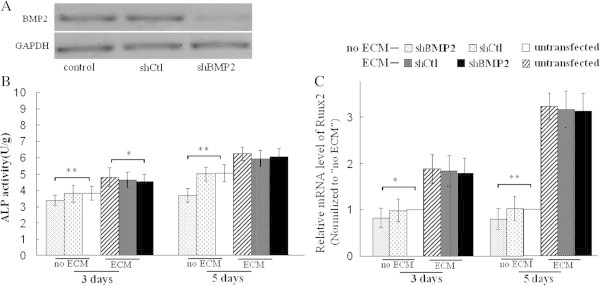
**Transfection with shBMP-2 affected ALP activity and Runx2 mRNA level of MC3T3-E1 cells re-seeded on osteoblast-ECM. A**, shBMP-2 reduced BMP-2 protein level of MC3T3-E1 cells. **B**, shBMP-2 lowered ALP activity of MC3T3-E1 cells seeded on cell culture plates without ECM coating (no ECM) , but had little effect on MC3T3-E1 cells seeded on osteoblast-ECM coated plates (ECM). **C**, shBMP-2 reduced Runx2 mRNA level, but had little effect on the cells seeded on osteoblast-ECM. ** P <0.01, *P <0.05, between the indicated groups, n = 5 per group.

Transfection with shBMP-2 reduced the ALP activity and Runx2 mRNA level of MC3T3-E1 cells seeded on plates without ECM coating after 3 and 5 days of growth. On the contrary, shBMP-2 had little effect on ALP activity and Runx2 mRNA level of MC3T3-E1 cells seeded on osteoblast-ECM coated plates (Figure 7). The results indicated knockdown of BMP-2 hardly affected on osteoblastic differentiation of MC3T3-E1 cells re-seeded on osteoblast-ECM.

**Figure 8:**
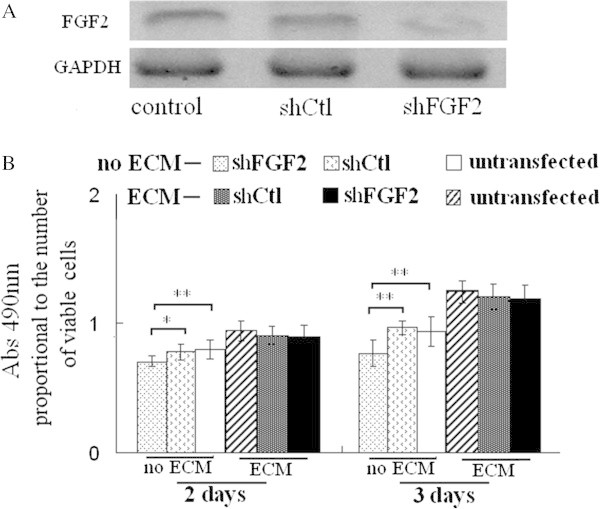
**Transfection with shFGF-2 affected proliferation of MC3T3-E1 cells seeded on fibroblast-ECM. A**, shFGF-2 reduced FGF-2 protein level of MC3T3-E1 cells. **B**, shFGF-2 lowered proliferative activity of MC3T3-E1 cells seeded on cell culture plates without ECM coating (no ECM), but had little effect on MC3T3-E1 cells seeded on fibroblast-ECM coated plates. ** P <0.01, *P <0.05, between the indicated groups, n = 5 per group.

The results of shFGF-2 were similar to shBMP-2, shFGF-2 reduced proliferative activity of the cells on no ECM-coated plates after 2 and 3 days of growth, and its effect on proliferative activity of the cells on fibroblast-ECM coated plates was very limited (Figure 8). The results indicated knockdown of FGF-2 had little effect on proliferation of MC3T3-E1 cells re-seeded on fibroblast-ECM.

## Discussion

ECM affects growth and differentiation of stem cells; it is one of the most important components of the stem cell niche (Metallo et al. 2007; Peerani and Zandstra 2010). BMSCs, the pluripotent stromal cells derived from bone marrow, can rapidly multiply billion-fold in a culture, and can be induced to differentiate into a variety of cell types, including osteoblasts (Prockop 1997; Jaiswal et al. 2000; Jiang et al. 2000). Considering multilineage differentiation potential of BMSCs and the microenvironment provided by ECMs for mammalian cells *in vitro*, the effect of different cell-derived ECMs (such as osteoblast-ECM and cardiac fibroblast-ECM) on the growth and differentiation of BMSCs *in vitro* is of the utmost importance.

ALP, BMP-2, osteopontin, Runx 2, OCN and extracellular calcium deposition, all were marker of osteogenic differentiation (Beck et al. 2000; Wu et al. 2009; Rider and Mulloy 2010; Mahalingam et al. 2011), so they were assayed to evaluate osteoblastic differentiation in our previous studies (Guo et al. 2011,2012). In this study, to investigate osteogenic differentiation of BMSCs seeded on ECMs, ALP activity, the levels of BMP-2 and osteopontin protein, mRNA expression of Runx2 and OCN, and extracellular calcium deposition were all analyzed.

We compared osteoblastic differentiation of BMSCs seeded on osteoblast-ECM and cardiac fibroblast-ECM. The activity of ALP and calcium deposition of BMSCs grown in the plates coated with osteoblast-ECM were higher than in other groups. BMSCs seeded on osteoblast-ECM had also the highest protein levels of BMP-2, osteopontin, and mRNA for Runx2 and OCN. These data demonstrate that osteoblast-ECM is more suitable for osteogenic differentiation than fibroblast-derived ECM.

However, in this study, the cardiac fibroblast-derived ECM had much stronger proliferative effect on BMSCs than osteoblast-derived ECM. Considering that the relative adhesion potentials of BMSCs were nearly same on the two ECMs, the results indicated that cardiac fibroblast-ECM had better proliferative effect than osteoblast-ECM. Compared with control group, the osteoblast-ECM also had proliferative effect.

BMP-2, accumulated in collagen-rich extracellular matrices produced by osteoblasts or in bone matrix, can induce differentiation of stem and mesenchymal cells into osteogenic cells capable of producing bone (Suzawa et al. 1999; Granjeiro et al. 2005). Covalently immobilized BMP-2 on a NHS-functionalized self-assembled monolayer, promotes the osteoblast phenotype in C2C12 cells (Pohl et al. 2012). FGF-2, an ECM-bound growth factor, binds to heparan sulfate glycosaminoglycan of ECM (Vlodavsky et al. 1987; Duchesne et al. 2012), the extracellular matrix–growth factor complexes play an important role in cell proliferation (Clark 2008). In this study, although knockdown of BMP-2 or FGF-2 inhibited osteoblastic differentiation or proliferation of MC3T3-E1 cells. The knockdown hardly effected osteoblastic differentiation or proliferation of MC3T3-E1 cells seeded on osteoblast-ECM or fibroblast-ECM. These results indicated osteoblast-ECM or fibroblast-ECM had enough bioactivity to induce osteoblastic differentiation or proliferation of MC3T3-E1 cells even the secretion of BMP-2 or FGF-2 was reduced significantly, at least in early time. It is probably that BMP-2 or FGF-2 in the two ECMs induces the differentiation or proliferation. In future study, we will investigate the probability.

Most components of the natural ECM have structural characteristics in nanometer dimensions, and the organization of cells and the corresponding tissue features are highly dependent on the ECM’s architecture Liu et al. (2009). For example, collagen is the major protein of the ECM, it arranges into nanofibers ranging from 50 to 500nm in diameter, its architecture plays a great role in cell behavior (Holzwarth and Ma 2011). This nanoscale fibrillar structure has been shown to be important for cell attachment, proliferation, and differentiation (Smith et al. 2009; Holzwarth and Ma 2011). In this study, the VG staining showed the collagen fibers in the two ECMs. In the future, we will investigate the relationship between bioactivity of ECM and the collagen’s architecture in nanometer dimensions.

In conclusion, the ECMs from two different sources present diverse bioactivity, the osteoblastic ECM has better osteoinductive potential and lower proliferative effect than fibroblastic ECM, knockdown of growth factors (BMP-2 or FGF-2) hardly influences differentiation and proliferation of re-seeded cells on the ECMs.

## Abbreviations

ECM: Extracellular matrix
BMP: Bone morphogenetic protein
OCN: Osteocalcin
Runx2: Runt-related transcriptional factor 2
FGF: Fibroblast growth factor
BMSCs: Bone marrow-derived mesenchymal stem cells
ALP: Alkaline phosphatase
GAPDH: Glyceraldehyde3-phosphatedehydrogenase
PCR: Polymerase chain reaction.
